# Supplementary material for: Hearing Problems in Indonesia: Attention to Hypertensive Adults
Source: Int J Environ Res Public Health. 2022 Jul 28;19(15):9222. doi: 10.3390/ijerph19159222 (PMC9367905; doi:10.3390/ijerph19159222)
Supplement: Supplementary file 1 [file ijerph-19-09222-s001.zip › ijerph-1765490-supplementary.pdf]

Table S1. Univariate logistic regression of hearing problem and demographic data based on gender

Predictors	Male				Female			
	P	Odds Ratio	95% CI Lower	95% CI Upper	P	Odds Ratio	95% CI Lower	95% CI Upper
Age group								
15-25 years old	0.121	0.621	0.34	1.135	0.029	0.514	0.282	0.935
26-35 years old	0.324	0.756	0.433	1.319	0.005	0.415	0.223	0.772
36-45 years old		1.0				1.0		
46-64 years old	0.021	1.77	1.089	2.876	0.072	1.55	0.961	2.499
Blood pressure								
AHA 2017								
Normal		1.0				1.0		
Elevated	0.46	1.231	0.709	2.139	0.257	1.432	0.769	2.665
Hypertension	0.174	1.37	0.87	2.157	0.197	1.307	0.87	1.962
INAHS 2019								
Non-Isolated systolic hypertension		1.0				1.0		
Isolated systolic hypertension	0.18	1.51	0.827	2.756	0.178	1.603	0.807	3.185
Hypertension medication								
Yes	0.008	3.422	1.379	8.492	0.000	4.746	2.582	8.724
No		1.0				1.0		
Hypertension self-report								
Yes	0.000	2.978	1.876	4.729	0.000	3.476	2.321	5.206
No		1.0				1.0		
Body Mass Index group								
Underweight	0.002	0.214	0.08	0.571	0.656	0.835	0.377	1.848
Normal	0.334	0.783	0.477	1.286	0.706	1.094	0.687	1.742
Overweight mild	0.383	0.726	0.354	1.49	0.767	1.099	0.588	2.053
Overweight severe		1.0				1.0		
Education								
No School	0.035	0.507	0.27	0.953	0.655	1.167	0.592	2.298
Senior high school or lower	0.112	0.643	0.373	1.109	0.713	0.886	0.465	1.689
Post senior high school		1.0				1.0		
Occupation								
Work	0.31	1.35	0.756	2.411	0.027	1.572	1.054	2.344
Not work		1.0				1.0		
General check-up								
Yes	0.002	2.12	1.326	3.389	0.000	2.636	1.599	4.343
No		1.0				1.0		
Outpatient care								
Yes	0.052	1.606	0.997	2.587	0.01	1.705	1.137	2.558
No		1.0				1.0		
Insurance ownership								
Yes	0.169	1.3	0.895	1.889	0.487	0.874	0.597	1.279
No		1.0				1.0		

AHA: American heart association; INASH: Indonesian society of hypertension

Table S2. Multivariate logistic regression analysis of hearing problem and demographic data based on gender

Predictors	Male				Female				Total Population			
	P	Odds Ratio	95% CI Lower	95% CI Upper	P	Odds Ratio	95% CI Lower	95% CI Upper	P	Odds Ratio	95% CI Lower	95% CI Upper
(Constant)	0.000	0.009			0.000	0.005			0.000	0.005		
Age*INASH-isosysht												
1 (15-25 y.o)*Non-isosysht	0.49	0.791	0.407	1.539	0.145	0.621	0.327	1.179	0.174	0.73	0.463	1.149
2 (15-25 y.o)*Isosysht	N/A	N/A	N/A	N/A	N/A	N/A	N/A	N/A	N/A	N/A	N/A	N/A
3 (26-35 y.o)*Non-isosysht	0.325	0.753	0.427	1.326	0.017	0.46	0.243	0.868	0.018	0.6	0.394	0.915
4 (26-35 y.o)*Isosysht	0.692	0.667	0.09	4.954	N/A	N/A	N/A	N/A	0.511	0.513	0.07	3.745
5 (36-45 y.o)*Non-isosysht		1.0				1.0				1.0		
6 (36-45 y.o)*Isosysht	N/A	N/A	N/A	N/A	0.484	1.684	0.391	7.256	0.62	0.698	0.169	2.887
7 (46-64 y.o)*Non-isosysht	0.218	1.392	0.823	2.353	0.303	1.323	0.777	2.252	0.076	1.401	0.966	2.032
8 (46-64 y.o)*Isosysht	0.01	2.62	1.26	5.449	0.989	0.994	0.417	2.371	0.064	1.692	0.969	2.954

Adjusted for all covariates (body mass index group, hypertension self-report, general check-up, outpatient care, education, occupation, insurance ownership)

AHA: American heart association; BP: Blood Pressure; *, Combine effect of age and blood pressure groups.
